# The Role of Self-Regulation in Forgiveness: A Regulatory Model of Forgiveness

**DOI:** 10.3389/fpsyg.2020.01084

**Published:** 2020-05-26

**Authors:** Man Yee Ho, Daryl R. Van Tongeren, Jin You

**Affiliations:** ^1^Department of Social and Behavioral Sciences, City University of Hong Kong, Kowloon, Hong Kong; ^2^Psychology Department, Hope College, Holland, MI, United States; ^3^Department of Psychology, Wuhan University, Wuhan, China

**Keywords:** forgiveness, emotion regulation, self-regulatory strength, self-control, self-regulatory fatigue

## Abstract

Forgiveness is an emotion regulation process that is important for both physical and mental health. Given its benefits, studying the facilitation of forgiveness is important. Researchers have already demonstrated the relationship between self-control and forgiveness. However, in this study, we aim to extend previous research by examining the regulating processes of forgiveness and the possible mediating role of emotion regulation in the relationship between self-regulatory strength and forgiveness. University students (*N* = 317) in Hong Kong who were recruited to participate in this study completed an online survey. The results of this study indicated that both self-regulatory strength and emotion regulation were significant predictors of forgiveness. Interestingly, cognitive reappraisal significantly mediated the association between self-regulatory fatigue and forgiveness. This suggests a potential self-regulation mechanism that leads to a prorelationship response and provides evidence for a regulatory model of forgiveness.

## Introduction

Forgiveness is a central feature of social life, helping to facilitate interactions between individuals and groups, as well as bolstering the functioning of committed, ongoing relationships (see Worthington and Wade, 2020 for a review). Empirical work has ranged from intergroup forgiveness (Van Tongeren et al., 2014) to interpersonal relationships (Fehr et al., 2010). One of the primary social benefits of forgiveness is its ability to preserve and restore valued relationships (e.g., Burnette et al., 2012). Though the conceptualizations of the forgiveness process have spanned the arenas of cognition, motivation, and behavior, the most consistent corpus of research has focused primarily on emotional processes. For example, researchers have examined the role of emotion in the forgiveness process in terms of emotion-focused coping strategies (Worthington and Scherer, 2004), emotional intelligence (Rey and Extremera, 2014), emotion regulation strategies in conflict resolution (Halperin, 2014), and physiological responses (Witvliet et al., 2001).

Previous research has demonstrated the relationship between emotion regulation and forgiveness (see Burnette et al., 2014 for a meta-analytic review), since forgiveness is often conceptualized as being rooted in emotions and requires a person to regulate their emotions toward the transgressor. Specifically, forgiveness is defined as the replacement of negative emotions (e.g., resentment, bitterness, anger, hatred, or hostility) toward a transgressor with positive emotions (i.e., empathy, sympathy, compassion, or love; Worthington et al., 2007). In other words, based on the *emotional replacement hypothesis*, forgiveness juxtaposes positive emotions against negative emotions; these positive emotions neutralize or replace all or part of the negative emotions (Worthington and Wade, 1999). However, such emotional transformations do not occur naturally or easily; individuals must overcome their natural tendencies to respond to an offender with anger and vengeance and instead engage in some form of regulation to respond positively. Accordingly, self-regulatory strength or emotion regulation looms large for many instances of the forgiveness process.

### Self-Regulatory Strength and Forgiveness

The ego-depletion model of self-regulation suggests that all forms of self-regulation draw on a common inner resource or limited pool of energy, called self-regulatory strength, which can be depleted. When individuals engage in an act of self-regulation that consumes considerable regulatory resources, subsequent self-control attempts can be impaired (Baumeister et al., 1998). Self-regulatory strength refers to the overall amount of self-regulatory capacity available to an individual pursuing a given goal, such as an interpersonal goal (Luchies et al., 2011). In short, when people engage in difficult tasks that require actively engaging the self and overriding a natural default behavior or sustained engagement in an arduous activity, the strain on one’s psychological resources can impair future endeavors that will require one to engage in similarly difficult processes.

However, the evidence regarding self-regulation, or, more specifically, ego-depletion, is mixed. For example, a preregistered study of the ego-depletion effect across multiple laboratories failed to replicate (Tangney et al., 2004; Hagger et al., 2016). Whether this failure to replicate is indicative of the lack of a reliable effect or another problem of the experimental design (e.g., imprecise tuning of the manipulation to the participant sample; see Baumeister, 2019; Caspi, 2000) remains to be seen; but it must be noted that the self-regulatory model in social psychology—and, by extension, within forgiveness processes—is not entirely undisputed and requires closer scrutiny. Finally, given that the experimentally manipulated ego-depletion effect is contested, work that employs nonmanipulated (i.e., self-reported) indices may help provide insights into these processes (though these have their own limitations, including the inability to draw causal conclusions).

Forgiveness may be one such instance of a process requiring self-regulatory strength; and, indeed, there has been some evidence of the importance of self-regulatory capability for forgiveness. Finkel and Campbell (2001) found that dispositional self-regulatory strength was positively associated with individuals’ accommodative tendencies (e.g., forgiveness) in romantic relationships, and that temporary self-regulatory fatigue decreased individuals’ likelihood of engaging in accommodative responses (e.g., forgiveness) to their partner’s destructive behaviors. Several other studies found that self-control predicted interpersonal success, higher relationship quality, greater relationship satisfaction, and fewer relationship conflicts from childhood to adulthood (Tangney et al., 2004; Luchies et al., 2011; Vohs et al., 2011). A meta-analytic review revealed that self-control had statistically robust association with small to moderate magnitude across 40 studies and 5,105 participants (Burnette et al., 2014). Building on this prior work, because forgiveness is an emotion regulation process that falls within the broader self-regulatory domain, we hypothesized that low self-regulatory strength (or high self-regulatory fatigue) is associated with lower forgiveness levels.

### Emotion Regulation and Forgiveness

Forgiveness is an emotion regulation strategy for coping with interpersonal conflict (Worthington and Wade, 2020). In particular, people may use emotion-focused coping when the perceived best way of dealing with an interpersonal transgression is to attempt to ameliorate immediate negative responses such as anger and hostility. In this way, this emotion regulation strategy requires self-regulatory strength, as it is one instantiation of a self-regulatory process. Following an offense, individuals may also seek to regulate their emotional experiences through emotion-focused coping strategies, such as self-soothing or avoidance (Worthington and Scherer, 2004).

Unforgiveness is theorized as a stress response to appraisals of interpersonal stressors, such as transgressions, betrayals, offenses, and wrongs (Berry et al., 2001). According to Lazarus and Folkman (1984) stress and coping model, an interpersonal transgression is an interpersonal stressor, and the forgiveness process is one way of reacting to, or coping with it.

Cognitive reappraisal is an antecedent-focused emotion regulation strategy that plays a vital role in reinterpreting an interpersonal harm (McCullough, 2001). Cognitive reappraisal involves transforming how an individual construes a situation in order to decrease its emotional impact (Gross, 1998). Forgiveness can function as a cognitive reappraisal process that eradicates anger, hostility, rumination, and their adverse effects in spite of feeling emotional pain and the desire for revenge (Worthington et al., 2007). Positive reappraisal of past negative events, such as reappraising the transgressor’s motivations in a benevolent manner (McCullough, 2001), is a key step in the forgiveness process. Therefore, in this study, we hypothesized that emotion regulation, especially cognitive reappraisal, is associated with higher forgiveness levels.

### The Mediating Role of Emotion Regulation

We see a gap in the existing forgiveness research regarding understanding how self-regulatory strength may operate through emotion regulation to impact the forgiveness process. Because forgiveness requires that individuals override their default, natural reactions to interpersonal offenses (i.e., unforgiveness) by instead regulating negative emotions and replacing them with neutral or positive emotions toward the offender, it seems that some degree of self-regulatory strength is necessary (see Burnette et al., 2014 for a review). To the degree that people have sufficient self-regulatory strength, they should be able to engage in emotion regulation strategies that facilitate forgiveness. Thus, these strategies are likely the mediating mechanisms by which self-regulatory capacity affects forgiveness.

According to the ego-depletion theory, an individual exercising self-control on one task who attempts to exert self-control on a second task simultaneously is more likely to fail due to overstrained resources (Geisler and Schroder-Abe, 2015). Individuals with low self-regulatory strength may be unable to exert self-control in regulating their emotions at all. Studies have shown that trait self-control was associated with successful regulation of negative emotions. Given the associations of self-regulatory strength and emotion regulation with forgiveness, we hypothesized that self-regulatory strength (or low self-regulatory fatigue) would be associated with forgiveness because such people would be better able to engage in emotion regulation (cognitive reappraisal). Thus, we predicted an indirect effects (mediational) model.

In this study, we investigated interpersonal forgiveness via emotion regulation and in consideration of individual differences in self-regulatory strength. Building on the consideration derived from the strength model of self-regulation, we propose a new regulatory model of forgiveness, in which emotion regulation (cognitive reappraisal) mediates the effect of self-regulatory strength (self-regulatory fatigue) on forgiveness.

## Methods

### Participants

The participants were 317 students (92 men, 218 women) at a university in Hong Kong. They were between the ages of 18 and 36 (*M* = 20.6 years, *SD* = 2.3). Most participants were single (65.8%), and a large number were in a relationship (25.4%). The majority of participants did not have any religious affiliation (60.2%). The rest identified as Christians (17.7%), Catholics (4.1), Buddhist (1.2%), or others (1.5%). Approximately half were majoring in humanities and social sciences (57.2%). The rest were studying commerce (12.4%), sciences and engineering (10.6%), creative media (4.9%), law (3.2%), veterinary medicine and life sciences (0.9%), and energy and environment (0.3%). Participants were recruited via various means, including recruitment posters displayed at university campuses, university-wide mailing list and in-class promotion. They participated in this study voluntarily. After they completed the study, they were automatically entered in a lottery for coffee coupons (HK$50, HK$100, HK$200) as an incentive.

### Procedure

Each participant completed an online questionnaire that assessed forgiveness, self-regulatory fatigue, and emotion regulation. Participants provided informed consent before participating in the study. This study was approved by the University Human Research Ethics Committee before it began.

## Materials

### Self-Regulatory Fatigue

The Self-Regulatory Fatigue Scale (SRF-S; Nes et al., 2013) was used to measure self-regulatory fatigue (the depletion of self-regulatory strength). The SRF-S consists of 16 items aimed at assessing participants’ self-regulatory fatigue. Sample items include, “I experience repeated unpleasant thoughts” and “I experience uncontrollable temper outbursts.” It employs a five-point Likert-type scale with responses ranging from 1 (*not at all true*) to five (*very true*); higher scores reflect chronic ego-depletion or a scarcity of self-regulatory resources. The SRF-S was shown to be a reliable and valid measurement in a Chinese sample (Wang et al., 2015) and demonstrated acceptable reliability in this study (Cronbach’s alpha = 0.70).

### Emotion Regulation

The Emotion Regulation Questionnaire (ERQ; Gross and John, 2003) was used to assess emotion regulation. The ERQ consists of 10 items that are assessed on a seven-point Likert-type scale to measures respondents’ tendency to regulate their emotions in two ways: (1) cognitive reappraisal and (2) expressive suppression. Example items include, “When I’m faced with a stressful situation, I make myself think about it in a way that helps me stay clam” (cognitive reappraisal) and “I control my emotions by not expressing them” (expressive suppression). Possible responses ranged from 1 (*strongly disagree*) to 7 (*strongly agree*). The Chinese version of the ERQ has been validated in a Chinese population (Zhang et al., 2014). The cognitive reappraisal and expressive suppression subscales have demonstrated acceptable reliability in the present study (Cronbach’s alphas = 0.86, and 0.72, respectively).

### Forgiveness

Forgiveness was assessed by the Trait Forgiveness Scale (TFS). The TFS consists of 10 items aimed at assessing participants’ self-appraisal of their proneness to forgive in interpersonal transgressions (Berry et al., 2005). Sample items include “I can forgive a friend for almost anything” and “I am a forgiving person.” Respondents rate each item on a five-point Likert-type scale from 1 (*strongly disagree*) to five (*strongly agree*). This scale has been reported as being reliable and valid across various studies (Berry et al., 2005). Two research assistants translated and back translated it into Chinese. The TFS demonstrated acceptable reliability in the present study (Cronbach’s alpha = 0.77).

A demographics questionnaire asking about the participants’ gender, age, marital status, religious beliefs, and academic major was also included in this study.

## Results

Descriptive statistics and zero-order correlations of study variables are shown in Table 1. The results of the correlation analysis indicated that the tendency to forgive was negatively associated with self-regulatory fatigue and positively correlated with cognitive reappraisal. Furthermore, self-regulatory fatigue was negatively associated with cognitive reappraisal and positively associated with expressive suppression. As expressive suppression was not associated with forgiveness, it was removed from all subsequent analyses.

**TABLE 1:** Descriptive statistics and correlations of study variables.

	***M(SD)***	**1**	**2**	**3**
1. Forgiveness	3.2 (0.6)			
2. Self-regulatory fatigue	3.0 (0.5)	−0.39**		
3. Cognitive reappraisal	4.9 (0.9)	0.31**	−0.32**	
4. Expressive suppression	16.3 (4.3)	−0.07	0.16**	0.01

**p < 0.01.

Correlation analyses were also conducted to examine the degree of associations between demographic variables (i.e., gender, age, marital status, religious beliefs, and academic major) and study variables. The results of Spearman’s rank correlation analyses showed that only religious beliefs were significantly associated with cognitive reappraisal and forgiveness. Therefore, the variable of religious beliefs (by using dummy coding) was statistically controlled for all subsequent analyses.

A hierarchical multiple regression analysis was performed to test the hypothesized mediation model. The religious beliefs variable was entered into the model in Step 1 to serve as a control. In Step 2, self-regulatory fatigue was added into the model as a predictor. In Step 3, cognitive reappraisal was entered into the model to test for a potential mediation effect. The results of the regression analysis indicated that religious beliefs (Christianity vs no religion) accounted for a significant amount of the variance in forgiveness (β = 0.13, *p* < 0.05). Self-regulatory fatigue accounted for a significant amount of the variance in forgiveness (β = −0.41, *p* < 0.001) and cognitive reappraisal (β = −0.33, *p* < 0.001). Cognitive reappraisal also accounted for a significant additional amount of variance in forgiveness (β = 0.17, *p* < 0.01) after controlling for self-regulatory fatigue. When cognitive reappraisal was included, the beta weight for self-regulatory fatigue decreased from −0.41 to −0.35 (*p* < 0.001), which suggests a partial mediation model (*R*^2^ = 0.21, *F*(1,280) = 12.40, *p* < 0.001) (see Table 2).

**TABLE 2:** Results of hierarchical multiple regression analysis on hypothesized model.

**Model**	**Predictors**	***R*^2^**	***R*^2^ change**	***F***	**β**
1		0.02	0.02	1.76	
	***Religion***				
	Buddhism				0.07
	Christianity				0.13*
	Catholicism				0.01
	Others				0.08
2		0.19	0.16	12.85***	
	***Religion***				
	Buddhism				0.09
	Christianity				0.16**
	Catholicism				0.04
	Others				0.06
	***SRF***				−0.41***
3		0.21	0.02	12.40***	
	***Religion***				
	Buddhism				0.08
	Christianity				0.13*
	Catholicism				0.04
	Others				0.07
	***SRF***				−0.35***
	***CR***				0.17**

SRF, self-regulatory fatigue; CR, cognitive reappraisal. *p < 0.05, **p < 0.01, ***p < 0.001.

**FIGURE 1:**
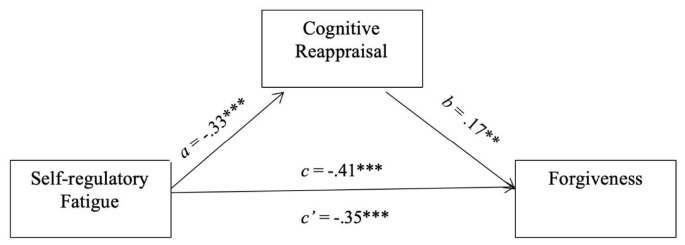
The regulatory model of forgiveness. The *c* and *c*’ indicate the total and direct effects from self-regulatory fatigue to forgiveness.

We further tested the significance of the indirect pathway from self-regulatory strength to forgiveness through the mediation of cognitive reappraisal using the PROCESS macro in SPSS. The bootstrapping procedure utilized 5,000 bootstrap samples and 95% bias-corrected confidence intervals (95% CIs). The exclusion of 0 from 95% CIs indicated a significant mediation effect (Hayes, 2013). The results of the bootstrapping analyses indicated that self-regulatory fatigue had exerted a significant direct effect on forgiveness, *c’* = −0.38, 95% CI [−0.50, −0.26] as well as a significant indirect effect on forgiveness through the mediator of cognitive reappraisal, *ab* = −0.07, 95% CI [−0.12, −0.02] (see Figure 1).

Alternate models were tested to eliminate two other possibilities: (1) a forgiving tendency might reduce self-regulatory fatigue, which might make individuals more likely to engage in cognitive reappraisals and (2) people who are generally good at cognitive reappraisal might be more likely forgive, which might then provide more self-regulatory strength. However, the results of hierarchical multiple regression analyses indicated that the *F* values were decreased, *F*(1,280) = 9.77, *p* < 0.001 in alternate model 1, and the beta coefficients of the key predictor variable were diminished in alternate model 2, which suggests that the alternate models did not fit the data better than the proposed mediation model.

## Discussion

In this study, we sought to expand our understanding of the conditions of transforming the motives to forgive by examining whether self-regulatory strength (self-regulatory fatigue) and emotion regulation (cognitive reappraisal) would be associated with the tendency to forgive among Hong Kong Chinese college students. Moreover, this study sought to uncover the regulating processes of forgiveness by investigating the mediating role of emotion regulation (via cognitive appraisal) in the association between self-regulatory strength and forgiveness.

The results of this study revealed that self-regulatory strength depletion (self-regulatory fatigue) was negatively associated with the tendency to forgive. That is, participants who reported lower levels of self-regulatory fatigue demonstrated a higher tendency to forgive others. This is consistent with Luchies et al. (2011) finding that self-regulatory strength has a significant positive impacts on close relationships. This finding supports the strength model of self-regulation in understanding interpersonal outcomes, such as forgiveness. The results of this study therefore indicate that chronic self-regulatory fatigue inhibits individuals’ tendency to engage in the transformation of prosocial motivations (i.e., forgiveness).

We also found that emotion regulation was positively associated with forgiving tendencies. Specifically, participants who regulated their emotions via cognitive reappraisal tended to engage in forgiveness. Our findings align with previous studies that linked cognitive reappraisal to a variety of positive outcomes within the domain of interpersonal relationships, such as positive relationships with others, higher peer rated relationship closeness, and greater peer-rated likability (Gross and John, 2003). Our findings may also point to the possibility that cognitive reappraisal may serve as an important self-regulatory process to transform the motivation of forgiveness.

Scientific research on the underlying processes between self-regulatory strength and forgiveness is lacking. In particular, whether emotion regulation plays a role in the relationship between self-regulatory fatigue and forgiveness remains unclear. The results of this study further elucidate the underpinnings of the transformation of prosocial motivation. Our findings indicate that cognitive reappraisal exerts additional effects beyond the effect of self-regulatory fatigue on forgiveness. More importantly, cognitive reappraisal mediate the negative relationship between self-regulatory fatigue and forgiveness. The findings of this study suggest the possibility that forgiveness may be an additive two-stage process through which individuals first inhibit destructive impulses by exercising their self-regulatory strength and then regulate their emotions via cognitive reappraisal. Both stages require separate exertions of self-regulation (self-regulatory fatigue and cognitive reappraisal). Self-regulatory depletion impairs the ability to engage in constructive cognitive processes, which in turn leads to lower forgiveness levels.

Interestingly, the results of this study showed that religious beliefs were positively associated with forgiveness. In particular, people who identified as Christian reported higher tendencies to forgive in comparison to those who had no religious affiliation. Forgiveness is one of the doctrines central to Christianity; therefore, it is possible that people who follow Christianity believed that they are supposed to forgive because they have been forgiven by God, and will thus be more prone to forgiving others. However, due to the unequal sample size of each religion, we need to be cautious in interpreting this result (see Davis et al., 2013 for a meta-analytic review on religion and forgiveness).

### Implications of the Present Research

A possible implication of the fact that self-regulatory fatigue was negatively associated with individuals’ ability to forgive others is that impulse regulation may be something of a double-edged sword. On the one hand, impulse regulation allows for thriving relationship functioning in that inhibiting destructive impulses promotes optimal interpersonal interaction. On the other hand, overregulating impulses can be problematic because such regulation depletes individuals’ capacity to regulate future impulses. Thus, attempts at constant, perfect self-control are likely to result in self-regulatory depletion or self-regulatory fatigue.

However, indiscriminate impulse indulgence is unlikely to improve relationship outcomes. The present study, for example, suggests that individuals are better able to forgive depending on the extent to which they exert emotional impulse regulation through constructive cognitive processes. Such prorelationship transformation of motivation benefits healthier relationship functioning. Perhaps the best way to achieve a compromise between impulse indulgence and regulation is to learn to recognize cues that indicate depletion (e.g., physical and emotional exhaustion) and to build up self-regulatory strength through repeated exercises (e.g., self-control exercises). Prior theoretical analyses and empirical evidence suggested that self-regulatory strength can be enhanced through such exercises and that exerting self-regulation tends to strengthen and improve our self-regulatory strength, much like weightlifting tends to increase muscular strength (Baumeister et al., 1994).

Emotion regulation can be used to downregulate negative emotions in the aftermath of an interpersonal offense. Emotion regulation involves complex cognitive processing. Cognitive emotion regulation refers to cognitive responses to emotional events that involve the attempt to alter individual emotional experiences, events and/or emotional types (Liu et al., 2019). Specifically, cognitive reappraisal is an effective emotion regulation strategy in which individuals change their understanding of emotional events by giving such events new meaning (McRae et al., 2012). Reframing potentially emotion-eliciting events (e.g., interpersonal transgressions) influences an individual’s willingness to forgive in an interpersonal context.

### Limitations and Strengths of the Present Research

We noted several limitations in the present research. First, we examined the tendency to forgive only in college students in Hong Kong. Therefore, the findings of this study may not be applicable to other populations (e.g., married couples) and other cultures (e.g., other Asian cultures). To test the generalizability of our findings, future research should examine the effects of self-regulation processes on forgiveness in other populations and in other cultures.

The use of the self-report method constituted another limitation. The results of this study might be influenced by socially desirable response tendencies, acquiescence bias, and the retrospective reconstruction of prior events. It is also difficult to interpret the results based on the criticism of self-report methodology. Although we used reliable and valid measure to assess people’s general propensity to forgive, forgiveness is not a one-size-fits all process—it takes all shapes and forms, and the magnitude of the offense is different from situation to situation. Thus, individual differences in forgiveness are to be expected. Consequently, future research should be conducted to incorporate behavioral or physiological measures of forgiveness and self-regulation in the context of ongoing interpersonal relationships.

This study also adopted a cross-sectional design, which may prevent us from ruling out reverse causalities and testing the multiple-stage self-regulatory processes of forgiveness. It is plausible that individuals high in trait forgiveness tend to preserve self-regulatory strength by disengaging from costly rumination and facilitating constructive cognitive reappraisal. Alternatively, it is also likely that individuals who are generally good at cognitive reappraisal tend to forgive due to less ego-depletion and more self-regulatory strength. Future studies should test the mediation model proposed in this study with a longitudinal design or a laboratory experimental design. Finally, we focused on trait-level variables, which may be less sensitive to temporal shifts than state-level variables. Thus, our findings speak to one’s general tendencies or dispositional patterns.

Despite the above limitations, an important strength of the present research is our adoption of a new approach to understand the self-regulation processes of forgiveness. This study demonstrated that self-regulatory fatigue is negatively associated with forgiveness via its association with cognitive reappraisal. This regulatory model of forgiveness indicates that high self-regulatory fatigue hampers cognitive self-regulation processing (cognitive reappraisal), which leads to less accommodative tendencies toward others.

## Conclusion

This study contributes to the existing literature by proposing a new regulatory model of forgiveness. This model facilitates the understanding of the regulatory processes of forgiveness in two ways: (1) direct pathways from both self-regulatory fatigue and cognitive reappraisal to forgiveness and (2) an indirect pathway from self-regulatory fatigue to forgiveness via cognitive reappraisal. This study furthers our understanding of how self-regulation processes influence the likelihood of forgiveness in an interpersonal context. Self-regulatory strength (i.e., self-regulatory fatigue) and emotion regulation (i.e., cognitive reappraisal) promote the transformation of prosocial motivations (i.e., forgiveness). The nuanced findings regarding the underlying mechanism of forgiveness from self-regulatory fatigue through cognitive reappraisal are more interesting. Consistent with our expectations, high self-regulatory fatigue weakens individuals’ ability to resist self-interested, instinctive reactions in favor of more personally costly, prorelationship responses (e.g., forgiveness) and this connection can be partly explained by their failure to engage in constructive cognitive processes.

## Data Availability Statement

The datasets analyzed in this manuscript are not publicly available for participants’ confidentiality reasons. Requests to access the datasets should be directed to my.ho@cityu.edu.hk.

## Ethics Statement

The studies involving human participants were reviewed and approved by City University of Hong Kong. The patients/participants provided their written informed consent to participate in this study.

## Author Contributions

MH has done most of the conceptual thinking, theoretical model building, the data collection and the data analysis. DV has offered conceptual framework on self-regulation process. JY has advised on model testing and statistical analysis.

## Conflict of Interest

The authors declare that the research was conducted in the absence of any commercial or financial relationships that could be construed as a potential conflict of interest.
